# High Prevalence of Porcine Circovirus 3 in Hungarian Pig Herds: Results of a Systematic Sampling Protocol

**DOI:** 10.3390/v14061219

**Published:** 2022-06-03

**Authors:** Barbara Igriczi, Lilla Dénes, Imre Biksi, Ervin Albert, Tamás Révész, Gyula Balka

**Affiliations:** 1 Department of Pathology, University of Veterinary Medicine, István Str. 2., 1078 Budapest, Hungary; igriczi.barbara@univet.hu (B.I.); denes.lilla@univet.hu (L.D.); biksi.imre@univet.hu (I.B.); albert.ervin@univet.hu (E.A.); 2 CEVA-Phylaxia (Ceva Sante Animale), 1107 Budapest, Hungary; tamas.revesz@ceva.com

**Keywords:** PCV3, prevalence, phylogeny, oral fluid, processing fluid, whole genome

## Abstract

Porcine circovirus type 3 (PCV3) is an emerging pathogen that has been reported worldwide in all ages of healthy and clinically ill pigs. The presence of this virus in Hungary has been confirmed in a commercial farm experiencing reproductive failures, but there were no data on the circulation of PCV3 in the country. Here we report the prevalence and the genetic diversity of PCV3 in Hungarian herds. To estimate the prevalence, 1855 serum samples, 176 oral fluid and 97 processing fluid samples were collected in a systematic, cross-sectional method from 20 large scale swineherds and tested by real-time qPCR. PCV3 was present in at least one type of diagnostic matrix in 19 out of the 20 (95%) pig farms. The highest detection rates were observed in the processing fluid samples (61%), but 41% of the oral fluid and 23% of the serum samples were positive. The virus was found in all age groups, and slightly more adult animals were infected than growing pigs, but the viral burden was lower amongst them. Phylogenetic analysis of nine complete genomes, obtained from either the sampled herds or organ samples of PCV3-positive carcasses, showed high nucleotide identity between the detected sequences, which all belonged to the PCV3a genotype. Our results indicate that PCV3 is widespread in Hungary, but in most cases, the virus seems to circulate subclinically, infecting all age groups and production phases without the presence of apparent clinical disease.

## 1. Introduction

Porcine circoviruses (PCVs) are small, non-enveloped single-stranded DNA viruses belonging to the *Circovirus* genus in the family of *Circoviridae*. Thus far, four types of PCVs have been identified. The first described member of the genus, the PCV1, was first reported in 1974 as a porcine kidney cell culture contaminant and is considered non-pathogenic for pigs [1,2]. However, PCV2 is responsible for significant economic losses worldwide. The virus was identified in the mid-1990s in Canada as the primary pathogen responsible for PCV2 systemic disease (PCV2-SD), formerly called postweaning multisystemic wasting syndrome (PMWS) [3]. Since its discovery, PCV2 infections were also linked with different clinical manifestations, including reproductive failure [4], porcine dermatitis and nephropathy syndrome (PDNS) [5] and respiratory and enteric disease [6,7]. As an emerging circovirus species, the PCV3 was identified in 2016 by next-generation sequencing methods in tissues of pigs suffering from PDNS, reproductive failure, myocarditis or multisystemic inflammation [8,9]. Recently, a novel, unclassified member of the genus was discovered and named PCV4. The newly described virus was detected with the aid of sequencing in pigs with severe respiratory and enteric disease in China [10] that had already been identified in Korea as well [11], but not in samples originating from Spain and Italy [12].

Since the first description of PCV3 in the United States, the presence of the virus has been reported in different countries in Europe [13,14,15], Asia [16,17] and America [18,19] in different ages of healthy and clinically ill pigs. Even though PCV3 is widespread globally and retrospective studies showed that it was circulating in pig populations long before its discovery [20], the exact role of PCV3 as a pathogen remains poorly characterized. PCV3 infection has been linked with several clinical-pathological conditions, such as swine respiratory and digestive disease [21,22], congenital tremors [23] or ear malformation and wasting disease [24,25], but the most frequently detected ones are reproductive failures [13,25,26,27] and multisystemic inflammation [8,24,25,26].

Further studies reported high loads of PCV3 within lesions of aborted fetuses and weak-born piglets and from cases of PDNS, pneumonia, periarteritis, myocarditis or encephalitis using in situ hybridization methods [8,24,25,26,28,29]. Recently two major disease outcomes were proposed to standardize diagnostic criteria for PCV3 associated disorders: the PCV3 reproductive disease in fetuses and sows (PCV3-RD) and the PCV3 systemic disease in pre- and postweaning pigs (PCV3-SD) [30].

In a recent study, six-week-old CD/CD piglets were inoculated with a PCV3 strain isolated from weak-born piglets or elevated stillborn and mummified fetuses. The animals were parallelly treated with keyhole limpet hemocyanin emulsified in incomplete Freund’s adjuvant for immune stimulation, and 28 days after challenge, they presented histological lesions consistent with multi-systemic inflammation characterized by myocarditis and systemic perivasculitis [31].

The genomic organization of PCV3 is very similar to PCV2, and their approximately 2000 nucleotides long circular genome consists of two major open reading frames (ORFs). The ORF1 encodes for replication-associated proteins, and the ORF2 encodes for the capsid protein. The amino acid identity of the PCV2 and PCV3 replicase proteins is only around 48%, and although their capsid proteins share some structural similarities, at the genetic level, there is only 27% amino acid identity [9,32]. Phylogenetic studies showed that PCV3 is distinct from PCV2, and it divides into two different clades (Clade 1—PCV3a and Clade 2—putative PCV3b genotype) based on the following criteria: bootstrap support higher than 90%, maximum genetic distance of 3% at the complete genome and 6% at the ORF2 levels [33].

The presence of the PCV3 in Hungary has been confirmed in a commercial farm experiencing reproductive failures [27]. We aimed to determine the prevalence and the genetic diversity of PCV3 in large-scale Hungarian pig farms and to compare the presence of the viral nucleic acid in different diagnostic materials, such as serum, oral fluid and processing fluids, assessing their utility in the diagnostics of PCV3.

## 2. Materials and Methods

### 2.1. Sample Collection

**Figure 1:**
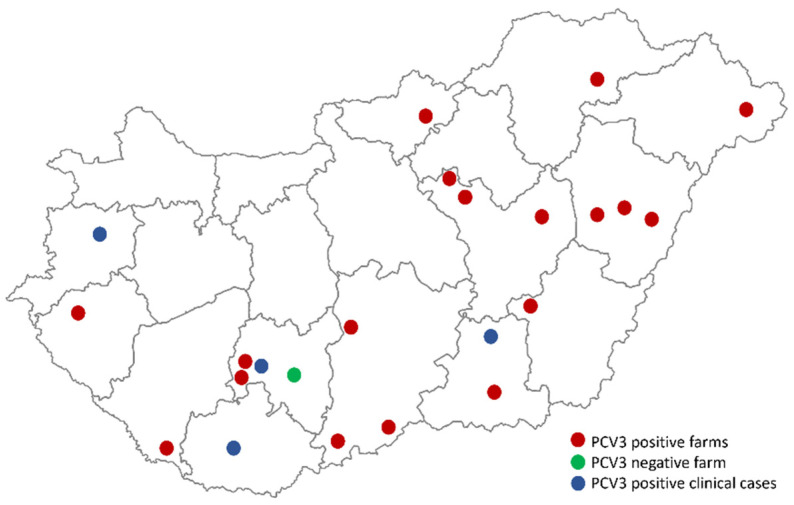
Map of Hungary showing the geographic location of the sampled farms (red/green) and the origin of PCV3-positive cases (blue) used for genome sequencing.

The samples analyzed in this study were collected from 20 Hungarian large-scale pig herds in 2020 and 2021 as a part of an active surveillance sampling (ethical permission number: PE/EA/544-5/2018) (Figure 1). The farms varied in sow herd size (from 520 to 2200), genetics and basic production parameters, but vaccination against PCV2 was performed in all of them. The participation of the different farms in the sampling campaign was voluntary regardless of their overall health status. We intended to cover every major swine producing area, but we did not decline anyone’s involvement, even if they were located in the same region or they were farms belonging to the same company. It can be stated, however, that no overt clinical disease was reported in the herds during the period of the samplings. 

On each farm, 10–10 blood samples were drawn from 2-, 4-, 6-, 8-, 10-, 14- and 18-week-old pigs, gilts, and sows of two and four parities. On six farms, we could not collect the full sample spectrum as not all age groups were present at the sampling time. Then, 5–five pen-based oral fluid samples were collected from weaned pigs (8–12 weeks of age, WOA) and fatteners (18–20 WOA), as well as five processing fluid samples, were obtained from each sow farm. The number of oral- and processing fluid samples also varied in some cases (Table 1). 

For oral fluid sample collection, a piece of cotton rope was hung in each pen for the pigs to chew on them. After 15–20 min, the ropes were removed and put in separate plastic bags, and then the liquid from each piece was squeezed into plastic tubes. Processing fluid samples were obtained during piglet castration. The testicles of the piglets were collected in plastic bags, and the liquid accumulated at the bottom was poured into plastic tubes. One plastic bag contained testicles and fluids of approximately 10 litters. Altogether, a total of 1855 serum samples, 176 oral fluid and 97 processing fluid samples were collected, and all were stored at −80 °C until further use. For the sake of full-length genome analysis, organ samples were obtained from carcasses that underwent diagnostic necropsies. The clinical signs and postmortem lesions were consistent with previously described PCV3 pathologies. Altogether, a total of five PCV3-positive organ pools originating from four farms were included in the study.

### 2.2. Sample Processing and DNA Extraction

Before the DNA extraction, equal volumes (100–100 µL) of serum samples were pooled by 5; thus, two pools corresponded to each age group. The processing and oral fluid samples were tested individually, and oral fluid samples were centrifuged shortly before extraction. The organ samples collected during the necropsies were homogenized by the use of Tissuelyzer II (Qiagen, Hilden, Germany) and centrifuged at 900× *g* for 3 min.

The DNA was extracted from 200–200 µL of pooled serum, oral fluid, processing fluid and tissue homogenate supernatants by QIAcube automatic nucleic acid extractor (Qiagen, Hilden, Germany) using the QIAmp cador Pathogen Mini Kit according to the manufacturer’s protocol. Nucleic acids were stored at −80 °C until further analysis.

### 2.3. Real-Time PCR Detection of PCV3

Real-time PCR (qPCR) was used to detect the presence of the viral DNA in the examined samples. The PCR assays were run on a Q qPCR Machine (Quantabio, Beverly, MA, USA), and each reaction contained 10 µL PerfeCTa qPCR ToughMix (Quantabio Beverly, MA, USA), 2 µL extracted DNA, previously published primers (900 nM) and probe (250 nM) [15] in a 20 µL final volume. We used the following temperature profile: 95 °C for 3 min followed by 45 cycles of 95 °C for 10 s and 60 °C for 30 s. Samples with Ct (cycle threshold) values higher than 38 were considered negative.

The statistical analysis of the qPCR results was performed using GraphPad Prism 8. The Ct values of different age groups and sample types were investigated using the Mann–Whitney test by pairwise comparison.

### 2.4. PCV3 Full-Genome Sequencing and Phylogenetic Analysis

Serum samples from PCV3-positive pools were extracted and tested individually. Full genome sequencing was attempted on samples with Ct values less than 30. The whole genome was amplified as three overlapping amplicons using Three sets of previously described primers and thermal conditions [15]. An amount of 5 µL extracted DNA was added to a mix of 5× Phusion^™^ GC Buffer (Thermo Scientific^™^, Waltham, MA, USA), 200 µM dNTPs, 1 µM of each primer and 0.5 units of Phusion^™^ High-Fidelity DNA Polymerase. The reactions were run in a Genesy 96T gradient PCR machine (Tianlong, China), and the PCR products were visualized by agarose gel electrophoresis. The amplicons with the correct length were manually cut and purified using the QIAquick Gel Extraction Kit (Qiagen, Hilden, Germany) according to the manufacturer’s protocols. To directly sequence the amplicons in both directions, the BigDye^™^ Terminator v3.1 Cycle Sequencing Kit (Thermo Fisher Scientific, Ljubljana, Slovenia) was used. Capillary electrophoresis was performed in a commercial sequencing laboratory at the Hungarian Natural History Museum.

All chromatograms were visualized and trimmed by Chromas 2.6.6 (Technelysium Pty Ltd., South Brisbane, Australia). The sequences were assembled and aligned using E-INS-I method of the online software MAFFT version 7 [34]. The phylogenetic trees were constructed using MEGAX [35] with the Maximum Likelihood method performing 1000 replicates of bootstrap analysis. A total of 41 reference sequences with different origins were downloaded from GenBank, aligned against our sequences and classified based on the method proposed by Franzo et al. [33]. The PCV3 sequences detected in this study were deposited into the NCBI GenBank with the accession numbers ON015882–ON015890.

## 3. Results

### 3.1. Detection Rates of PCV3 in Serum, Oral- and Processing Fluid Samples in Different Pig Farms

PCV3 was detected in 19 of the 20 (95%) examined herds in at least one type of diagnostic matrix (Figure 1). The viral DNA was identified in all three sample types on nine farms. The virus was detected in either two types of sample materials or just in oral fluid samples on the other farms. The different diagnostic matrices showed different prevalence rates in the positive farms: 5% to 71% PCV3 positivity was observed in the serum pools, 10–90% in the oral fluid samples, and 20–100% prevalence in the processing fluid samples (Table 1). The highest detection rates were observed in the processing fluid samples, as the overall proportion of PCV3-positive samples was 61% in the positive farms. The detection frequencies were significantly lower in the oral fluid samples, as the virus was detected in 40% of the samples. The lowest PCV3 detection rate was observed in the serum pools, as the viral DNA was found only in 23% of the samples (Table 1).

Ct values of the positive serum pools and oral fluid samples were relatively high considering the viral quantities. Of the 84 positive serum pools and 70 oral fluid samples, only three pools and five oral fluid samples had Ct values less than 30. The mean Ct value of the positive samples was 34.02 ± 2.56 in the case of the serum samples and 34.60 ± 2.42 in the oral fluids.

**Figure 2:**
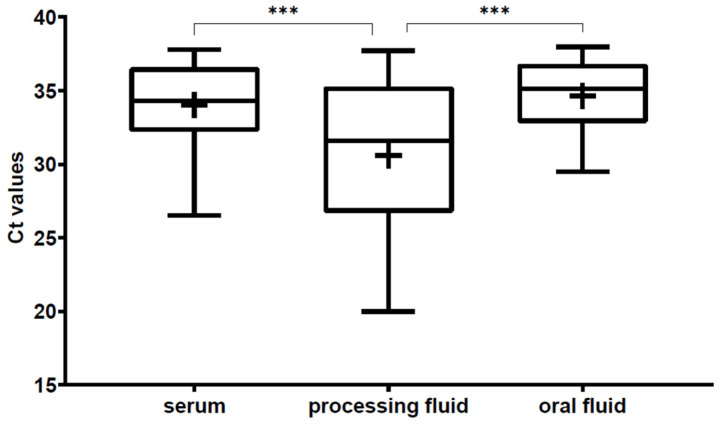
Boxplots representing the Ct values of the PCV3-positive samples of the three different sample types. The whiskers show the minimum and the maximum, and the “+” signs represent the average. The horizontal lines of the box show the first quartile, the median and the third quartile. The statistical comparison of the Ct values was performed using the Mann–Whitney test. The asterisks above the boxes represent the statistically significant differences (***: *p* < 0.001).

The Ct values of the processing fluid samples were significantly lower than the other two diagnostic materials, with a mean value of 30.18 ± 5.09 (Figure 2). Of the 56 positive processing fluid samples, 27 had Ct values between 20 and 30.

### 3.2. PCV3 Detection in Different Age Groups

**Figure 3:**
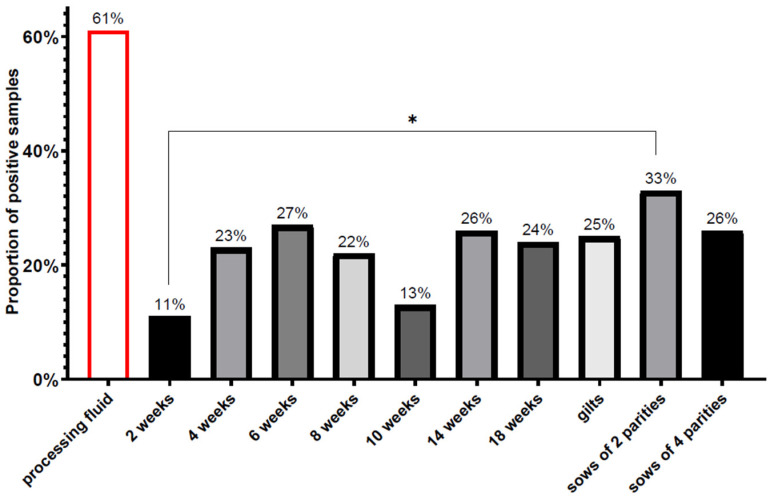
Percentages of PCV3-positive processing fluids and serum sample pools of different age groups. The statistical comparison of the PCV3 prevalence of each age group was performed by Fisher’s exact test. The asterisk above the columns represents the statistically significant difference (*: *p* < 0.05).

**Figure 4:**
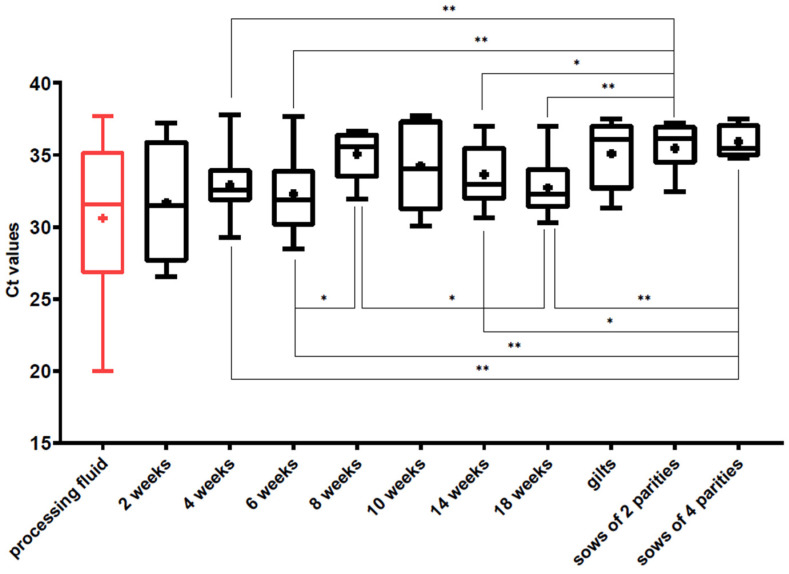
Boxplots representing the Ct values of the PCV3-positive processing fluids and serum sample pools of different age groups. The whiskers show the minimum and the maximum, and the “+” signs represent the average. The horizontal lines of the box show the first quartile, the median and the third quartile. The statistical comparison of the Ct values was performed using the Mann–Whitney test. The asterisks above the boxes represent the statistically significant differences (*: *p* < 0.05; **: *p* < 0.01).

PCV3 was found in every age group examined, and the average prevalence of the virus in the serum sample pools of the different age groups ranged from 11% to 33% (Figure 3). The lowest detection rate was observed in two-week-old pigs, whereas one-third (33%) of the sample pools of sows of two parities were positive. On average, the presence of the viral DNA was detected in 17% of the suckling piglets (11% of 2 WOA, 23% of 4 WOA) serum samples, 20% of the weaned pigs (27%, 22% and 13% of 6, 8 and 10 WOA, respectively) samples and 25% of the fatteners’ (26% and 24% of the 14 and 18 WOA) serum samples, 25% of the gilts’ samples and 30% of the sows’ (33% of two parities, 26% of four parities) samples. The mean Ct values of the PCV3-positive serum samples of different age groups ranged from 31.69 ± 4.42 (2 weeks old piglets) to 35.91 ± 1.17 (sows of four parities). Low Ct values indicating higher viral DNA copy amounts were more frequent in younger pigs and a slight increase was observed in the mean Ct values in the older age groups (Figure 4). Besides the serum samples of the suckling piglets, the lowest Ct values were found in the processing fluid samples representing the youngest age group, the newborn piglets. Pen-based oral fluid samples represented groups of weaned piglets of 10–12 WOA and fatteners of 18–20 WOA where the PCV3 detection rates were 50.63% and 35.44%, respectively.

### 3.3. PCV3 Circulation Patterns in Three Different Farms with High PCV3 Prevalence

**Figure 5:**
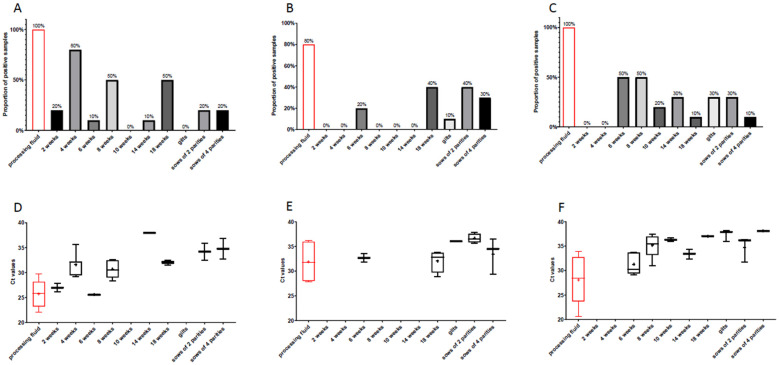
(**A**–**C**) Percentages of PCV3-positive processing fluid and serum samples of different age groups from Farm K (**A**), Farm JA (**B**) and Farm SZ (**C**). (**D**–**F**) Boxplots representing the Ct values of the PCV3-positive processing fluid and serum samples of different age groups from Farm K (**D**), Farm JA (**E**) and Farm SZ (**F**). The whiskers show the minimum and the maximum, and the ”+” signs represent the average. The horizontal lines of the box show the first quartile, the median and the third quartile.

As the within-herd, cross-sectional prevalence rates of the sampled herds were quite different, we chose three farms—with relatively high prevalence rates and all examined age groups available—to examine samples of positive serum pools individually. We found several differences in the patterns of PCV3 circulation on these farms (Figure 5). On Farm K, the virus already appeared in 2-week-old piglets and amongst all age groups, the detection rate was the highest in the 4-week-old piglets (80%), but half of the 8- and 18-week-old pigs’ samples were also positive (Figure 5A,D). On the other hand, on Farm JA older age groups showed higher prevalence, especially the 18 weeks old pigs and sows of 2 parities where 40% of the serum samples were positive (Figure 5C,E). On Farm SZ the virus appeared in 6-week-old piglets, and a relatively high detection rate (50%) was observed in the 6- and 8-week-old age groups. The prevalence on this farm seemed to decrease towards the older age groups (Figure 5C,F). Interestingly, the detection rates in processing fluid samples were equally high on Farm K and SZ, as all samples were positive, and Farm JA also showed high (80%) PCR positivity rates. The percentage of PCV3-positive oral fluid samples on Fam K, JA and SZ were 50%, 89% and 70%, respectively.

### 3.4. Genetic Analysis

For genome sequencing, individual samples with the lowest Ct values were selected from positive serum pools as well as organ samples obtained from PCV3-positive clinical cases. Altogether we were able to obtain the full genome in nine cases. The length of the genomes was 2000 nt long, as expected, and comparative nucleotide sequence analysis revealed 98.8–99.85% identity between them.

**Figure 6:**
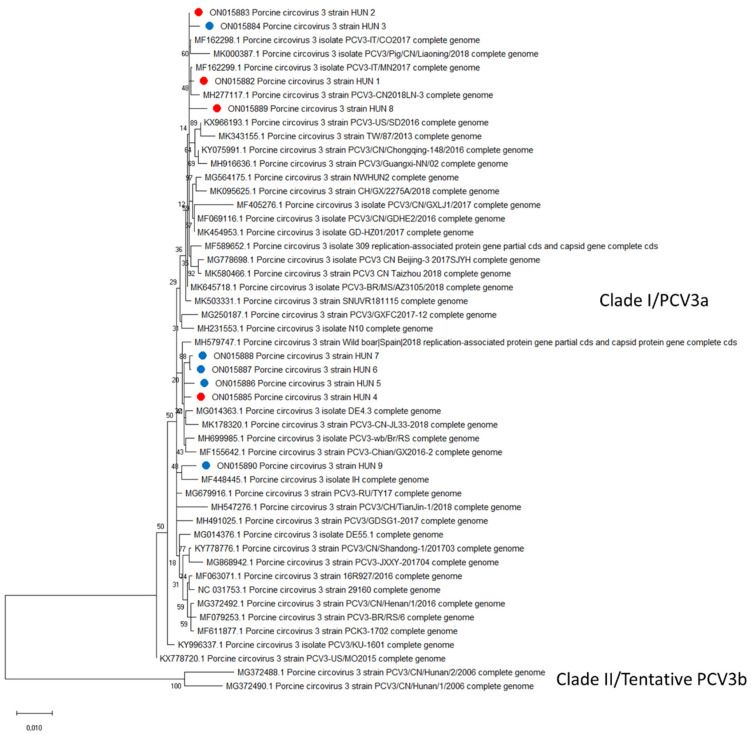
Phylogenetic analysis of PCV3 full-genome sequences. The phylogenetic tree was constructed using MEGAX [35] with the Maximum Likelihood method performing 1000 replicates of bootstrap analysis. A total of 41 reference sequences listed by Franzo et al. [33] were aligned against the sequences detected in this study. The sequences obtained from serum samples collected on the examined farms are presented by red dots. The blue dots mark the sequences obtained from PCV3-positive clinical cases.

The classification of these strains was based on a comparison with 41 PCV3 reference sequences. According to the genotypic classification method proposed by Franzo et al. [33], all PCV3 strains can be clustered into two clades. All nine of our sequences belonged to Clade I (PCV3a genotype), and their homology with the previously described sequences ranged from 89.95% to 99.95% (Figure 6). Although most of our sequences were nearly identical and clustered together, the position of the strains on the phylogenetic tree did not reflect the geographical relations of the different herds of origin. The phylogenetic analysis also revealed a very close relation to Italian, Chinese and Korean PCV3 strains (99.7%, 99.85% and 99.2% nucleotide identity, respectively).

## 4. Discussion

PCV3 is an emerging pathogen that has been reported in many countries all over the world. The exact pathogenic role of the virus is still under debate. PCV3 infection has been connected with different clinical-pathological conditions, but several studies reported that the virus also circulates in clinically healthy farms [14,15,36,37,38]. In 2019, Deim et al. reported the presence of the virus in a Hungarian pig farm suffering from reproductive disorders [27]. Besides this case, there were no data about the presence of the PCV3 in other parts of the country. Our study aimed to assess the prevalence of PCV3 in the Hungarian pig population.

Our results showed that PCV3 is widespread in Hungary, as its nucleic acid was detected in 19 of the 20 farms involved in our study. Altogether 23% of the serum pools (85/371), 40% of the oral fluid samples (71/176) and 61% of the processing fluid samples (59/97) were positive for PCV3. The detection rates in serum pools are similar to those observed in Poland (25%) [14] and Denmark (30%) but slightly higher than the values reported in Italy (18.18%), Spain (14.89%) [15] or Slovenia (13.1%) [39].

PCV3 detection in oral fluid samples has been reported in several studies. This non-invasive sampling method is quick, easy, and cost-efficient, and the samples are reliable representations of a whole pen. Similarly to our results (40%), a Korean study reported a PCV3 prevalence of 44.2% in oral fluid samples [36], and Woźniak et al. detected the virus in 37.3% of the oral fluid samples gathered from different Polish herds [40]. On the other hand, Chinese researchers described lower PCV3 prevalence (12.3%) [41], while a study from Slovenia reported a very high detection rate (73%) in this type of sample [39].

Using processing fluids to monitor the presence of different infectious agents has a lot of well-known advantages and it has been used for the detection of various porcine pathogens, including PRRSV [42], APPV [43] or *Mycoplasma hyopneumoniae* [44]. A retrospective study from the USA commonly detected PCV3 in clinical cases of PDNS and PMWS and tissue samples from aborted fetuses. Several different sample types, including serum, oral fluids and processing fluids, were tested for PCV3 by PCR, and the proportions of positive samples (14% of serum, 39% of oral fluid and 60% processing fluid samples) were similar to what we detected in this study [45]. To our knowledge, this is the first prospective study in a peer-reviewed paper where processing fluid samples were used for PCV3 detection and prevalence estimation by comparing them to other diagnostic matrixes. The high detection rates observed suggest that using processing fluids is a convenient way to verify the presence of PCV3 in a given herd and to monitor PCV3 infection in newborn piglets, where the virus is reported to be involved in various pathologies including respiratory disorders, diarrhea, abortions, early death and myocarditis as well as neurological disorders [8,24,26,28,29]. Presence in this age group also suggests active virus circulation among sows and gilts and transplacental infection.

Recently published studies have shown the presence of PCV3 in pigs of all ages. A longitudinal study from 2019 also concluded that the virus infects pigs from all production phases and seems to cause a long-term infection [46]. Several reports from different countries examined the within-herd dynamics of the infection, but it’s not easy to compare them as the sampling methods, and the age or health status of the examined animals are different. In Japan, the authors found the highest PCV3 prevalence in suckling piglets (13.3%), followed by fatteners (9.4%) and weaned pigs (9%) [47]. On the other hand, in a Polish study, PCV3 was reported in more than one-quarter of samples obtained from weaners, fatteners or sows (26.1%, 28% and 29%, respectively), while the samples obtained from suckling piglets only showed 5% PCV3 prevalence [14]. Franzo et al. also reported an increasing PCV3 detection frequency from piglets to weaners, but it seemed to decline in older animals [48].

Our study focused mainly on monitoring the presence of PCV3 in every age group of the examined herds. The sampling method used here covers almost all age groups and sow parities. Pooling the individual serum samples and using a pen- and litter-representative oral- and processing fluid samples allows us to gain information on the viral burden of numbers of animals at once in a rather cost-effective way. Our results show that PCV3 can be found in all age groups, and there are no significant differences in the prevalence of the virus among them, except in the comparison of the 2-week-old piglets and the sows of 2 parities. Altogether 17% of the suckling piglets (11% of 2-week-old, 23% of 4-week-old pigs) and 20% of the weaned pigs (27% of 6-week-old, 22% of 8-week-old, 13% of 10-week-old pigs) serum sample pools were positive for PCV3. The low detection rate in the 2-week-old age group might be linked with the protective role of maternal immunity, and then the slight increase in the 4- and 6-week-old animals might relate to the waning of the protection. Moreover, it is well-known that the process of weaning is a dramatic stress factor for the piglets and leads to immunological, intestinal or behavioral changes [49,50]. Therefore, it is possible that during the first weeks after weaning, the pigs are more susceptible to infections, as they also encounter increased amounts of pathogens while co-mingling with non-littermate piglets [51]. 

The decreasing proportions of the PCR’s positive samples in the 8- and 10-week-old pigs might suggest that the sampled animals mount protective adaptive immunity and overcome the infection. In the older age groups, 25–25% of the fatteners (26% of the 14-week-old and 24% of the 18-week-old pigs) and gilts’, and 30% of the sows’ (33% of sows of 2 parities, 26% of sows of 4 parities) serum sample pools were PCV3-PCR-positive. The increased detection rates in the 14-week-old pigs could be related to the regrouping and mixing of the animals in the fattening pens.

Interestingly, the Ct values of the positive samples of the different age groups seemed to have an increasing trend towards the older animals indicating decreasing amounts of viral copies. Pairwise statistical comparisons showed that the Ct values of the samples obtained from sows were significantly higher than those of the suckling piglets, weaners or fatteners. Based on our data, it seems like there are more adult animals infected than growing pigs, but the viral burden is lower in this age group. We found no significant difference between the Ct values of the PCV3-positive serum pools and the oral fluid samples, but the Ct values of the processing fluid samples were significantly lower than the other two materials. Although the Ct values of the serum sample pools of the 2-week-old pigs were the lowest among the serum samples, the PCV3 prevalence in this age group is relatively low (11%), suggesting that there are just a few animals born with relatively high levels of viraemia. 

It is important to mention that according to our protocol, the serum samples were representing 10 animals per age group in the given farms; the processing fluid samples, however, corresponded to approximately 10–15 litters with the male piglets only (tail docking was not used in most of the farms) resulting in 35–50 times more newborn animals sampled on farm level compared to one age group of serum samples. The high prevalence and the relatively low Ct values might be related to the vertical transmission and/or the colostral shedding of the virus, as has already been described [29,52]. 

On the other hand, our results may be explained by the protective role of maternally derived immunity, considering that the highest prevalence was observed among the serum samples of the sows and the lowest among the suckling (2–4-week-old) piglets. Similarly to PCV2, there might be an age-dependent susceptibility [53] to PCV3 infection and/or within-host replication linked to the declining levels of maternally derived antibodies, but further virological and serological studies are needed to understand the role of passive immunity and seroconversion in the case of PCV3 infections.

To gain more information on the viral circulation on pig farms, individual serum samples from three different herds were analyzed. Our results showed that PCV3 infects every age group, but some interesting differences were observed comparing the patterns detected on each farm (Figure 4). On farm K, the prevalence of the virus in sows and gilts was lower than in the other two farms: altogether, 13.3% of sampled gilts and sows were PCV3-positive, and the Ct values of the positive samples were relatively high, indicating low levels of viraemia and minimal virus circulation in the reproductive herd. Even though antibody levels were not measured in our study, it can be speculated that seroprevalence was similarly low in sows and gilts, resulting in the transfer of inadequate levels of maternal antibodies to piglets, which allows the virus to spread quickly among the suckling piglets. The latter could have resulted in the high percentage (80%) of the 4-week-old viremic pigs (all processing fluid samples were also positive on this farm). 

On farm JA and SZ, the virus was absent from suckling piglets and only appeared in 6-week-old weaned pigs. On both farms, the prevalence of PCV3-positive samples in gilts and sows was higher than on farm K (26.7% on farm JA and 23.3% on farm SZ). It is plausible that the maternally derived immunity protected the animals until weaning on these two farms, and the virus could only circulate in older age groups. Interestingly, the processing fluid samples collected from both farms were positive for PCV3 (80% on farm JA and 100% on farm SZ).

Based on these data, the main question is, is there increased pre-weaning mortality if the piglets are PCV3 infected? That would mean that some of those infected piglets died before reaching 2 weeks of age when the serum samples for this study were taken. Further studies are needed to confirm whether PCV3 positivity at birth indicates a correlation with early deaths of newborn animals. Our further goal is to investigate dead suckling piglets for the presence of PCV3 and for pathologies that are already connected with the infection.

Since the discovery of PCV3, different classification methods have been proposed. Recently Franzo et al. defined two different PCV3 clades after analyzing the broadest dataset of the available PCV3 genomes [33]. According to their method, all nine of our sequences clustered within the PCV3a genotype, with high nucleotide identity between each other and the corresponding reference sequences as well. This close phylogenetic relation suggests a much slower mutation rate compared with PCV2. The long-lasting worldwide PCV3 circulation reported by retrospective studies also suggests a fairly high viral stability [20,54,55].

The limitation of our research is that there was no differentiation between apparently healthy and diseased farms/animal groups, as the samples were not submitted as diagnostic material but rather as a part of an active, intentional survey performed to evaluate the overall prevalence within-herd infection dynamics of PCV3. No evidence of a significant disease outbreak was reported from the participating farms during the sampling campaign. Our further research will focus on the connection between various pathologies and the presence/infectious burden of the virus.

## 5. Conclusions

This study confirms that PCV3 infection is widespread in the Hungarian pig population. By comparing the different diagnostic matrixes, the viral DNA is more commonly detected in processing fluid samples, than in serum or oral fluids. Although the virus seems to infect all age groups and circulate subclinically in the sampled herds, there were important differences in the detection rates and circulation patterns between farms. Further studies on PCV3-positive farms are warranted to determine the role of PCV3 infection in the proposed disease outcomes.

## Figures and Tables

**Table 1:** Summary of the examined Hungarian farms, sample types and sample sizes. The number and proportion of the PCV3-positive samples are also included in the table. The grey cells correspond to sample types that were not collected.

	Serum				Processing Fluid			Oral Fluid		
Farms	Sample Size	Number of Pools	Positive Sample Pools	Proportion of Positive Sample Pools	Sample Size	Positive Samples	Proportion of Positive Samples	Sample Size	Positive Samples	Proportion of Positive Samples
Farm B	100	20	1	5%	5	4	80%	10	1	10%
Farm C	100	20	1	5%	4	2	50%	10	3	30%
Farm D	100	20	0	0%	10	0	0%	10	5	50%
Farm F	100	20	0	0%	5	5	100%	10	4	40%
Farm H	130	26	6	23%	2	1	50%	10	3	30%
Farm HM	100	20	5	25%	5	1	20%	10	0	0%
Farm HO	100	20	0	0%	5	1	20%	10	1	10%
Farm HS	85	17	5	29%	3	3	100%	0	0	0%
Farm J	80	16	9	56%	5	5	100%	8	7	88%
Farm JA	100	20	7	35%	5	4	80%	9	8	89%
Farm K	100	20	11	55%	5	5	100%	10	5	50%
Farm KU	60	12	4	33%	5	1	20%	10	0	0%
Farm N	60	12	2	17%	4	4	100%	4	0	0%
Farm O	100	20	1	5%	8	0	0%	10	5	50%
Farm P	100	20	7	35%	5	5	100%	10	6	60%
Farm PR	70	14	10	71%	2	0	0%	10	9	90%
Farm S	100	20	3	15%	5	5	100%	10	0	0%
Farm SO	100	20	2	10%	5	4	80%	10	7	70%
Farm SZ	100	20	11	55%	9	9	100%	10	7	70%
Farm Z	70	14	0	0%	0	0	0%	5	0	0%
**Total**	**1855**	**371**	**85**	**23%**	**97**	**59**	**61%**	**176**	**71**	**40%**

## Data Availability

PCV3 full genome sequences analyzed in our study are available at the NCBI GenBank with the accession numbers ON015882–ON015890.
